# Medicinal fungus *Phellinus igniarius* alleviates gout *in vitro* by modulating TLR4/NF-kB/NLRP3 signaling

**DOI:** 10.3389/fphar.2022.1011406

**Published:** 2022-10-21

**Authors:** Xuebin Zhou, Qiyuan Shi, Jinhua Li, Shengli Quan, Xinyue Zhang, Lili Gu, Hongxing Li, Yue Ju, Min Hu, Qin Li

**Affiliations:** School of Pharmacy, Hangzhou Medical College, Hangzhou, ZheJiang, China

**Keywords:** medicinal fungus Phellinus igniarius, anti-gout, metabolic mechanism, TLR4, NF-kB, NLRP3 inflammasome

## Abstract

**Background:** *Phellinus igniarius* (*P. igniarius*) is a valuable medicinal and edible fungus with various biological activities such as anti-inflammation, antioxidation, and immune regulation. In this study, we explored the effects of *P. igniarius* on a gout model *in vitro*.

**Methods:** The DPPH, ABTS, and FRAP methods were combined to determine and compare the antioxidant activities of wild *P. igniarius* total polyphenols (WPP) and cultivated *P. igniarius* total polyphenols (CPP) *in vitro*. Spectrophotometry was used to compare the inhibitory effect of WPP and CPP on xanthine oxidase (XO) activity to evaluate anti-hyperuricemia activity *in vitro*. HUVECs were stimulated with monosodium urate (MSU) crystals for 24 h to establish an acute gouty inflammation model *in vitro*. The protective effects were compared by measuring cell viability; the contents of ICAM-1, IL-1β, IL-6 and VCAM-1; the protein expressions of TLR4 and NLRP3; reactive oxygen species production; and the nuclear translocation of NF-κB p65. UHPLC-QE-MS technology was used to explore the potential metabolic mechanism of *P. igniarius* against gout.

**Results:** WPP and CPP had strong antioxidant capacity, and the antioxidant capacity of CPP was similar to that of WPP. In a comparative experiment of xanthine oxidase activity inhibition by WPP and CPP, the IC_50_ values were 88.19 μg/ml and 108.0 μg/ml, respectively. At a dose of 40 μg/ml, WPP and CPP significantly improved the decrease in cell viability induced by monosodium urate (150 μg/ml) and inhibited the increase in inflammatory factors such as ICAM-1, IL-1β, IL-6, and VCAM-1. The increase in TLR4 and NLRP3 protein expression induced by MSU crystals in HUVECs was also significantly inhibited by total polyphenols from wild and cultivated *P. igniarius*. In addition, both significantly improved MSU-induced ROS overproduction and NF-κB p65 nuclear translocation. WPP and CPP may primarily be involved in phenylalanine metabolism and lysophosphatidylcholine metabolism in their role in the treatment of gout.

**Conclusion:** CPP and WPP both showed good antioxidant activity and xanthine oxidase inhibitory activity and had good therapeutic effects on the gout model *in vitro*. Furthermore, this study indicated that cultivated *P. igniarius* had a protective effect similar to that of wild *P. igniarius*, which would be expected to improve the shortage of wild *P. igniarius* and promote the development of the cultivated *P. igniarius* industry and product development.

## Introduction

Gout is a clinically common metabolic disease, which is a major problem impacting human health (Stack et al., 2019; Ashiq et al., 2021). Gout is a disorder of purine metabolism in the body, leading to high concentrations of uric acid in the blood and deposition of sodium urate crystals in the joints, which induces an inflammatory response that manifests as the pathogenesis of gouty arthritis as the main disease, affecting the quality of life of patients (Ghaemi-Oskouie and Shi, 2011; Singh and Gaffo, 2020; Yin et al., 2020).

An increase in serum urate concentrations (hyperuricemia, ≥7.0 mg/dl) manifests as inflammation induced by the deposition of MSU crystals in cartilage, synovial bursa, tendons, or soft tissues (Garcia et al., 2016). MSU crystals are known to activate TLR4, a key factor in the initiation of neuroinflammation, and TLR4 expression further activates and phosphorylates NF-κB (Zeng, 2019). NF-κB, a downstream target protein of TLR4 (Yin et al., 2021), is activated to promote the release of a range of inflammatory factors (Guo et al., 2020), such as TNF-α, IL-6 (Fan, 2017), IL-1β and others (Borstad et al., 2004). Normally, IκBα and NF-κB exist in the cytoplasm as an inactive dimer. After phosphorylation of IκBα by IκB kinase, phosphorylated NF-κB translocates from the cytoplasm to the nucleus, thus inducing transcriptional activation of NLRP3 inflammatory vesicles and increased expression of inflammatory factors. NLRP3 inflammasomes produce reactive oxygen species (ROS) during activation, but the sources of these ROS and the mechanism by which NLRP3 participates in their production have not been clarified.

Given the intensity of the inflammatory reactions that characterize an acute attack, oral colchicines and nonsteroidal anti-inflammatory drugs (NSAIDs) are appropriate first-line drugs for this phase. However, oral colchicines and NSAIDs have adverse effects such as severe gastrointestinal reactions and toxicity (Borstad et al., 2004; Sabina et al., 2011). Therefore, the need to identify new anti-inflammatory treatments for gouty arthritis has stimulated many recent studies to focus on natural products (Ahmad et al., 2008) such as herbs (An et al., 2010), as people urgently need anti-gout drugs.

*P. igniarius*, a well-known mushroom belonging to the family Polyporaceae, is distributed in many East Asian countries such as China, Korea, and Japan (Mao, 2000; Dong et al., 2019) and is commonly referred to as “*Sanghuang*” in China (Dong et al., 2019). Its main components include polysaccharides, flavonoids, polyphenols, steroids, and organic acids, and it therefore has good anti-inflammatory, antioxidant and antitumor activities (Ding et al., 2016; Wang et al., 2018; Gu et al., 2019; Luan et al., 2022). Many studies have shown that it has good anti-uric acid activity, but the specific mechanism has not been clarified (Liu et al., 2019; Xing et al., 2021).

In conclusion, we hypothesize that *P. igniarius* may down-regulate MSU crystal-induced gout-related inflammatory factor expression in HUVECs through mediating the TLR4/NF-κB/NLRP3 signaling pathway. In this study, MSU was used to induce HUVECs to produce an acute gouty inflammation model *in vitro* to study the anti-inflammatory effects of wild and cultivated *P. igniarius* and further explore the specific mechanism of its inhibitory effect through enzyme kinetics and metabolomics studies. In addition, the medicinal value of wild *P. igniarius* is high, but the supply is insufficient and therefore the price is high, which has limited the development and utilization of *P. igniarius* as a medicinal fungal resource. Fortunately, artificial cultivation is effective. However, there are few comparative studies on wild and cultivated *P. igniarius*. Another purpose of this study was to compare and evaluate the efficacy of wild *P. igniarius* and cultivated *P. igniarius* in the treatment of hyperuricemia and gouty arthritis and to promote its industrial development and product development.

## Materials and methods

### Plant material

The Zhejiang Qiandao Lake Sangdu Edible Fungus Professional Cooperative sold us wild *P. igniarius* samples (Hangzhou, China). The *Phellinus igniarius* that was artificially cultivated was a strain known as “Zhehuang No. 1,” which was also supplied by Zhejiang Qiandao Lake Sangdu Edible Fungus Professional Cooperative. The Horticulture Institute of Zhejiang Academy of Agricultural Sciences and the Institute of Edible Fungi of Shanghai Academy of Agricultural Sciences both recognized it as *Phellinus igniarius*. All samples were kept in a dim setting with constant humidity and temperature.

## Preparation of polyphenols from P. igniarius

Response surface approach was used to identify the ideal conditions for the extraction and purification of polyphenols from *P. igniarius* by referring to the polyphenol concentrations and adhering to scientific standards and environmental protection protocols. In particular, the *P. igniarius* dried fruit body was crushed and put through a 40-mesh filter. The powder was extracted with 16-fold 70% ethanol for 2 h at 80°C in the dark after being steeped in 70% ethanol for 30 min. Under the same circumstances, the residue was removed once more. An ethanol extract of both wild and cultivated *P. igniarius* was produced by combining these two filtrates and removing the solvent under negative pressure at 60°C without light. After that, the crude extract solution was adsorbed by an HP20 macroporous resin, contaminants were cleaned up with 10% ethanol using five times the volume of the solution, and then the solution was eluted with 40% ethanol using five times the volume of the solution. The eluates were gathered, pooled, and then turned into a powder by freeze-drying. WPP and CPP were created in succession, stored at -20°C, and kept in the dark. The amount of total polyphenols was calculated using the Folin-Ciocalteu technique.

## Comparison of total antioxidant capacity between WPP and CPP *in vitro*

### Determination of total antioxidant capacity by the DPPH method

Aliquots of 2.5, 5, 10, 20, 40 and 80 μg/ml Vc, WPP or CPP 133.3 μl were added to a 200 μl DPPH working solution (Jiancheng, Nanjing, China). As a control, 2.5, 5, 10, 20, 40 and 80 μg/ml Vc, WPP or CPP 133.3 μl were added to 200 μL 80% ethanol. The blank tube contained 200 μl of 80% ethanol and 300 μl of the DPPH working solution. The absorbance (A) was measured at 517 nm after mixing and incubation at room temperature for 30 min. The DPPH free radical clearance rate (%) = [1- (A_sample_- A_control_)/A_blank_] × 100%. The IC_50_ value was calculated using a logistic regression curve.

### Determination of total antioxidant capacity by the FRAP method

Aliquots of 2.5, 5, 10, 20, 40 and 80 μg/ml Vc, WPP or CPP 5 μl were added to 180 μl of FRAP working solution (Jiancheng, Nanjing, China) with four wells each. Distilled water was added to three wells as a blank control. A standard curve was drawn with FeSO_4_ as the standard. Absorbance (A) was determined at 593 nm after incubation at 37°C for 5 min. The absorbance measured for each sample was compared with the standard curve, and the concentration equivalent to the FeSO_4_ standard solution was used to represent the antioxidant capacity. The FRAP was used to compare the total antioxidant capacity of the sample and the positive control: 1 unit FRAP = 0.5 mM FeSO_4_. The smaller the FRAP, the higher the total antioxidant capacity.

### Determination of total antioxidant capacity by the ABTS method

Aliquots of 2.5, 5, 10, 20, 40 and 80 μg/ml Vc, WPP or CPP 10 μl, and 20 μl enzyme application solution were added to 170 μl of ABTS working solution (Jiancheng, Nanjing, China) with four wells each. Distilled water was added to three wells as a blank control. A standard curve was drawn with Trolox as the standard. The absorbance (A) at 405 nm was determined after incubation at ambient temperature for 6 min. The absorbance measured for each sample was compared with the standard curve, and the concentration equivalent to the Trolox standard solution was used to represent the antioxidant capacity. The total antioxidant capacity of the tested samples and the positive control were compared by ABTS, with 1 unit ABTS = 0.5 mM Trolox. The smaller the ABTS, the higher the total antioxidant capacity.

### Comparison of inhibition of XO activity *in vitro* between WPP and CPP

Four groups were established: a blank control group, a positive control group, the WPP group and the CPP group. The WPP group and the CPP group were treated with a drug solution at different concentrations, with four wells per concentration. PBS was added to the blank group, and an allopurinol solution was added to the positive control group at different concentrations, with four wells for each concentration. The XO solution (Yuanye, Shanghai, China) was added to the wells of each group, and incubated at 37°C with an enzyme label. The XA solution (Yuanye, Shanghai, China) was then added to each well, and the absorbance (A) was measured immediately at 295 nm and read every 1 min. The change value was recorded for 6 min, and the XO inhibition rate (IR) and half-maximum inhibitory concentration (IC_50_) were calculated.

IR(%)=[(dAdt)blank−(dAdt)sample]/(dAdt)blank×100% (1)

The reaction time was chosen based on variations in absorbance, and in this formula, dA represents the absorbance difference from the time the reaction started and when it finished. The IR was calculated from the average values of the four wells. The IC_50_ value was calculated using a logistic regression curve.

### Cell culture and treatment

HUVECs (Bogoo Biological Technology, Shanghai, China) were grown in DMEM (Gibco, United States) culture medium containing 10% FBS (Siji Green, Zhejiang, China), 100 units/ml penicillin, and streptomycin at 37°C in a humidified atmosphere with 5% CO_2_. The cells in the logarithmic phase were subcultured and tested.

### MTT assay

MTT was used to detect cellular activity. The HUVEC monolayers were digested with pancreatin (Solarbio, Beijing, China), and then the HUVECs were plated in a 96-well plate and allowed to attach overnight. Next, the HUVECs were treated with different concentrations of MSU (Yuanye, Shanghai, China), WPP and CPP. The supernatant was discarded after incubation for 24 h, and 20 μl of MTT solution was added. The supernatant was discarded after incubation for 4 h, and 150 μl of DMSO was added with oscillation for 10 min. The OD was measured at 490 nm:

$$Cell\;Viability\left(\%\right)=\left({OD}_{drug}-{OD}_{control}\right)/\left({OD}_{blank}-{OD}_{control}\right)\times 100\%$$ (2)

### Determination of ICAM-1, VCAM-1, IL-1β and IL-6 in cell culture medium by ELISA

HUVECs were plated in a 6-well plate and allowed to attach overnight before they were treated with different concentrations of WPP or CPP and MSU for 24 h. The culture medium of each group was then extracted. The levels of ICAM-1, VCAM-1, IL-1β and IL-6 in the cell culture medium were determined with an ELISA kit (Mlbio, Shanghai, China).

### Western blot analysis

The cells were lysed in ice-cold RIPA buffer, and cell lysates were clarified by centrifugation, diluted in 2× SDS loading buffer, resolved by SDS‒PAGE and transferred onto PVDF, which was subsequently blocked with 5% skimmed milk. The membranes were then incubated overnight at 4°C with primary antibodies followed by incubation with horseradish peroxidase-conjugated antibodies. The immunoreactive protein bands were visualized with an enhanced chemiluminescence (ECL) kit (New Cell & Molecular Biotech, Suzhou, China).

### DCFH-DA fluorescent probe assay

The cell culture medium was withdrawn, the DCFH-DA was diluted with PBS (Biosharp, Anhui, China), and the cells were then given two PBS washes. The cells were then added to the diluted DCFH-DA solution, and they were incubated at 37°C. To completely eliminate any DCFH-DA that did not reach the cells, PBS was applied three times to the cells. Following the addition of PBS, the fluorescence was observed, captured on camera, and its average optical density was measured and quantitatively analyzed using ImageJ software. The excitation and emission wavelengths were 485 nm and 528 nm, respectively.

### Immunofluorescence assay

The cell culture medium was discarded, PBS (Biosharp, Anhui, China) was used to wash the cells three times, a 4% paraformaldehyde solution (Solarbio, Beijing, China) was added to fix the cells, the fixative was removed, and an immunostaining blocking solution was added at room temperature. The primary antibody was incubated overnight at 4°C, and goat anti-rabbit IgG H&L (Alexa Fluor^®^ 488) was diluted at 37 °C. A blocking solution (including DAPI) was added. The cells were observed under a fluorescence microscope.

## UHPLC-QE-MS for cell metabolomics analysis

### Cell sample preparation

The cells in each group were removed from the culture medium, quickly washed with precooled PBS solution, digested with trypsin, and then added to the culture medium. The medium was gently shaken to stop digestion of the infiltrated cells. With rapid counting, 1 × 10^7^ cells per bottle were taken as samples (i.e., 1 unit), quickly placed in a precooled 5 unit quencher containing 60% methanol (Macklin, Shanghai, China) and 8.5 g/L ammonium bicarbonate, adjusted to pH 7.4 with 12 M hydrochloric acid, and placed in a slightly oscillating centrifuge tube for 10 s. The mixture was allowed to cool at -20°C, centrifuged at 4°C and 1,000 × g for 1 min, and then the supernatant was removed, and the cells were placed in liquid nitrogen for 30 s and preserved at -80°C.

### Metabolite extraction

For lyophilization, the materials were moved to a 2 ml EP tube. 200 L of water were then added to the samples. The samples were vortexed for 30 s before being frozen with liquid nitrogen and thawed three times. The samples were sonicated in an ice-water bath for 10 min. The protein concentration was assessed using 50 μl of homogenate. The leftover amount was then mixed with 600 L of acetonitrile:methanol (1:1) and put to a 2 ml EP tube. The samples were vortexed for 30 s, then incubated at -40°C for 1 h, and then centrifuged for 15 min at 4°C at 12000 (RCF = 13800 g, R = 8.6 cm) rpm. The supernatant was transferred to an EP tube in an amount of 660 L, and it was dried in a vacuum concentrator. After drying, a proportionate amount of acetonitrile, methanol, and water (2:2:1) with an internally labeled standard mixture was added. The samples were vortexed for 30 s before being sonicated for 10 min in an ice-water bath. The materials were then centrifuged for 15 min at 4°C at 12000 (RCF = 13800 g, R = 8.6 cm) rpm. A new glass vial was used to transfer the resultant supernatant for analysis. An equivalent quantity of the supernatant was mixed to create the quality control (QC) sample.

### LC‒MS/MS analysis

A UHPLC system (Vanquish, Thermo Fisher Scientific) with a UPLC BEH Amide column (2.1 mm 100 mm, 1.7 m) connected to a Q Exactive HFX mass spectrometer was used to perform the LC-MS/MS analysis (Orbitrap MS, Thermo). 25 mmol/L each of ammonium acetate and ammonia hydroxide in water (pH = 9.75) (A) and acetonitrile made up the mobile phase (B). The injection volume was 2 L, and the autosampler’s temperature was 4°C. Because it can acquire MS/MS spectra in information-dependent acquisition (IDA) mode under the control of the acquisition software, the QE HFX mass spectrometer was chosen (Xcalibur, Thermo). The acquisition program constantly assesses the complete scan MS spectrum in this mode. The following parameters were adjusted for the ESI source: spray voltage of 3.6 kV (positive) or -3.2 kV, sheath gas flow rate of 30 arb, aux gas flow rate of 25 arb, capillary temperature of 350°C, full MS resolution of 60000, MS/MS resolution of 7,500, collision energy of 10/30/60 in NCE mode (negative).

### Data annotation, multivariate statistical analysis and metabolic pathway analysis

The raw data was programmatically converted to the mzXML format and then ordered to perform metabolic annotations. This information included peak detection, extraction, alignment, and integration. For logarithmic conversion and centralized formatting, the final datasets were entered into the SIMCA V15.0.2 software program (Sartorius Stedim Data Analytics AB, Umea, Sweden). Principal component analysis (PCA) and orthogonal projections to latent structures-discriminate analysis (OPLS-DA) were carried out in succession to depict group separation and identify significantly altered metabolites. The OPLS-DA was used to calculate the VIP value, or variable importance in the projection.

For the purposes of the following study, metabolites between the two groups that had P 0.05 (Student’s t test) and VIP >1 were regarded as differential metabolites. Utilizing volcano plots, the up- and downregulation of various metabolites was demonstrated. To identify the key metabolic pathways with the highest correlation, the differential metabolites were subjected to pathway enrichment analysis using the KEGG database and MetaboAnalyst (http://www.metaboanalyst.ca/), which further revealed the related metabolic mechanism of wild and cultivated *Phellinus igniarius* in the treatment of hyperuricacidemia.

### LC-MS/MS data

The original data of HPLC chromatogram has been uploaded to the public database “MetaboLights”, and the original data can be downloaded according to the database number MTBLS5795.

### Statistical analysis

GraphPad Prism V8.0.2 software was used for statistical analysis and data visualization. Multiple comparisons between groups were performed using a one-way ANOVA or two-way ANOVA. The data are expressed as the mean ± standard deviation (SD), and a *p* < 0.05 was considered statistically significant.

## Results

### The total antioxidant capacity of CPP is similar to that of WPP

**FIGURE 1:**
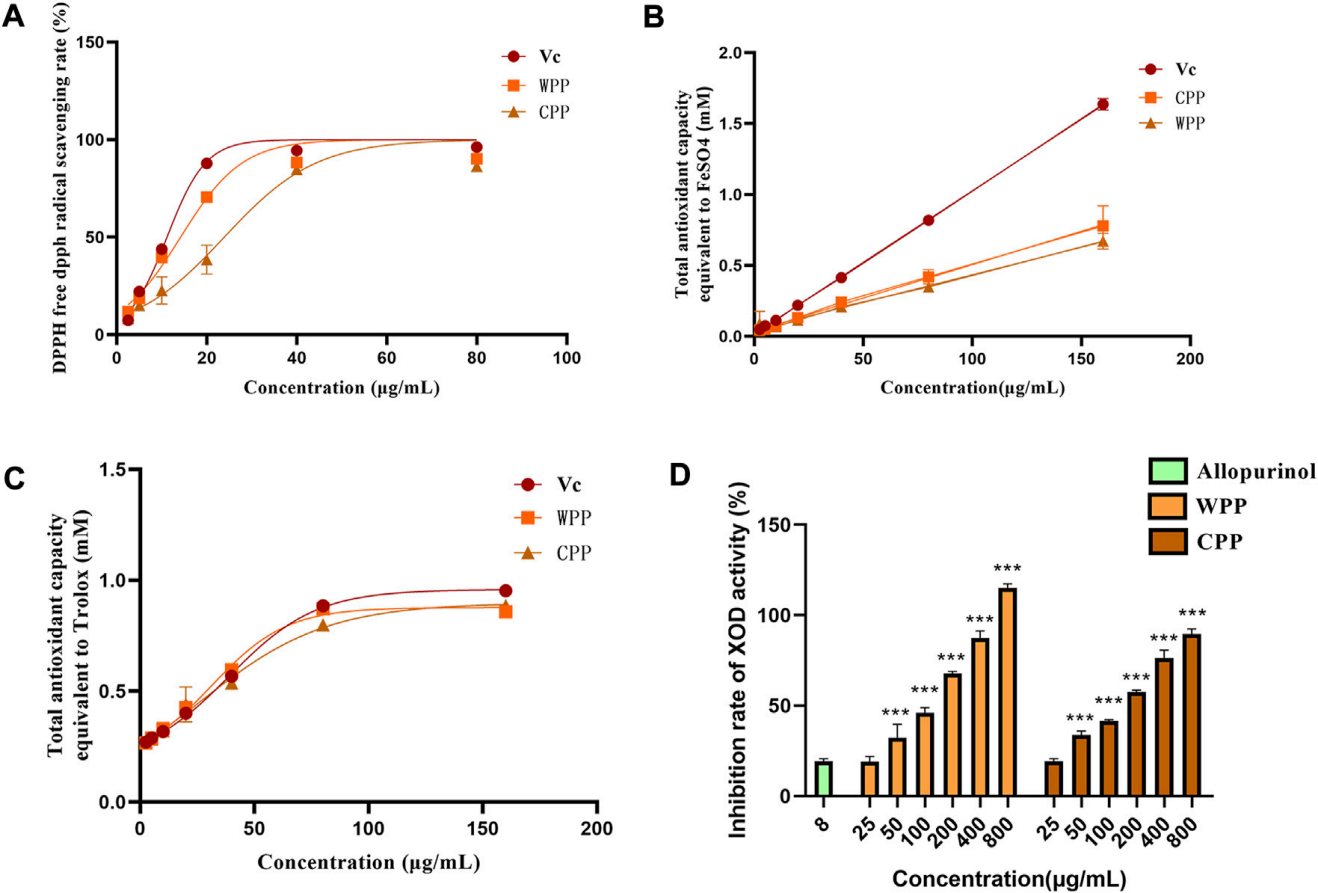
Comparison of DPPH free radical scavenging rates between WPP and CPP **(A)**, comparison of FRAP values between WPP and CPP **(B)**, and comparison of ABTS values between WPP and CPP **(C)**. Comparison of inhibitory effects of WPP and CPP on XO, ^***^ *p* < 0.001 vs. allopurinol (8 μg/ml), *n* = 4 **(D)**.

With vitamin C (Vc) as the positive control, the total antioxidant capacities of WPP and CPP were detected using the DPPH, FRAP and ABTS methods. As shown in Figure 1, 1A, the IC_50_ values of Vc, WPP and CPP for DPPH radical scavenging were 9.994 μg/ml, 12.28 μg/ml and 21.00 μg/ml, respectively. The FRAP values (Figure 1B) of Vc, WPP and CPP were 47.83 μg/ml, 98.75 μg/ml and 116.97 μg/ml, respectively. The ABTS values (Figure 1C) of Vc, WPP and CPP were 39.69 μg/ml, 40.44 μg/ml and 44.40 μg/ml, respectively. In conclusion, WPP and CPP have a strong total antioxidant capacity, and the total antioxidant capacity of CPP is similar to that of WPP.

### The inhibitory effect of CPP on XO activity was similar to that of WPP

As shown in Figure 1D, with allopurinol (8 μg/ml) as the control, WPP and CPP significantly inhibited XO activity and showed dose-dependent tolerance within a certain concentration range. The XO inhibition of both in the dose range of 50–800 μg/ml was statistically significant (*p* < 0.001). The IC_50_ values of WPP and CPP for the inhibition of XO activity were 88.19 μg/ml and 108.0 μg/ml, respectively. The ability of CPP to inhibit XO activity was similar to that of WPP.

### WPP and CPP attenuated the decrease in cell viability and cell cytotoxicity caused by MSU in HUVCECs

**FIGURE 2:**
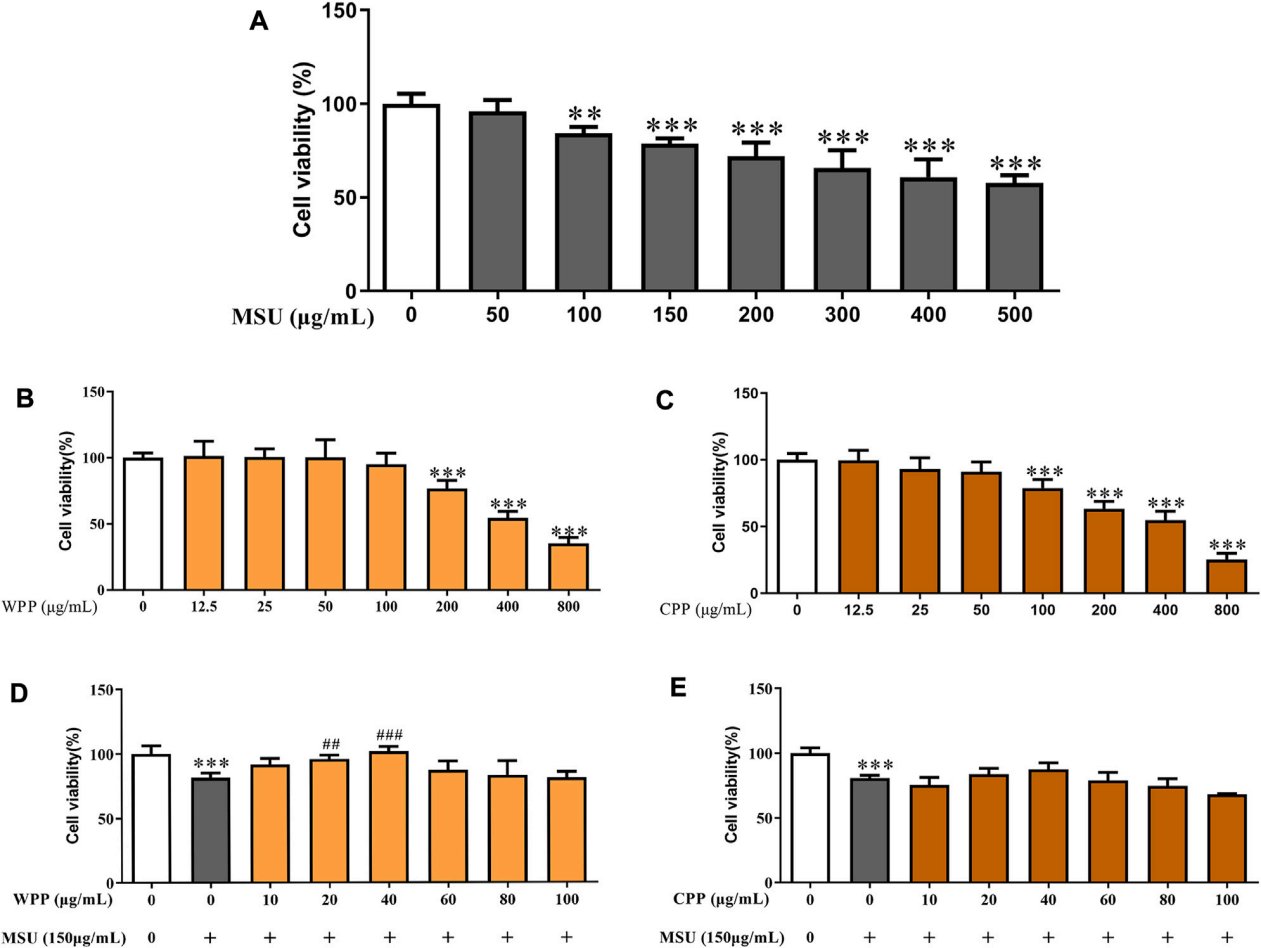
The effects of MSU on cell viability, *p < 0.05, **p < 0.01, ***p < 0.001 vs. the control group, *n* = 6 **(A)**. Cell viability of HUVECs treated with different concentrations of WPP as determined by the MTT assay, ***p < 0.001 vs. the control group, *n* = 6 **(B)**. Cell viability of HUVECs treated with different concentrations of CPP as determined by the MTT assay, ***p < 0.001 vs. control group; *n* = 6 **(C)**. Protective effect of WPP on MSU-induced HUVEC injury, ***p < 0.001 vs. control group; ^##^ *p <* 0.01, ^###^ *p* < 0.001 vs. positive group, n = 6 **(D)**. Protective effect of CPP on MSU-induced HUVEC injury, ***p < 0.001 vs. the control group, *n* = 6 **(E)**.

To evaluate the toxicity of MSU, WPP and CPP, HUVECs were treated with different concentrations of MSU (0–500 μg/ml), WPP or CPP (0–800 μg/ml for both) for 24 h, and cell viability was evaluated by the MTT assay. As shown in Figure 2, 2A, MSU concentrations above 150 μg/ml had an extremely significant inhibitory effect on cell viability. WPP and CPP had no significant cytotoxic effect on cells below 200 μg/ml (Figure 2B) and 100 μg/ml (Figure 2C), respectively. We chose these concentrations for the following experiments. To evaluate the protective effects of WPP and CPP on MSU-induced cell death, HUVECs were treated with MSU and WPP or CPP for 24 h. At a concentration of 40 μg/ml, both WPP and CPP reduced the loss of cell viability induced by MSU (Figures 2D,E). In summary, WPP and CPP at a dose of 40 μg/ml were selected for subsequent studies on efficacy and mechanism.

### WPP and CPP can reduce the production of inflammatory factors in an acute gouty inflammation model *in vitro*

**FIGURE 3:**
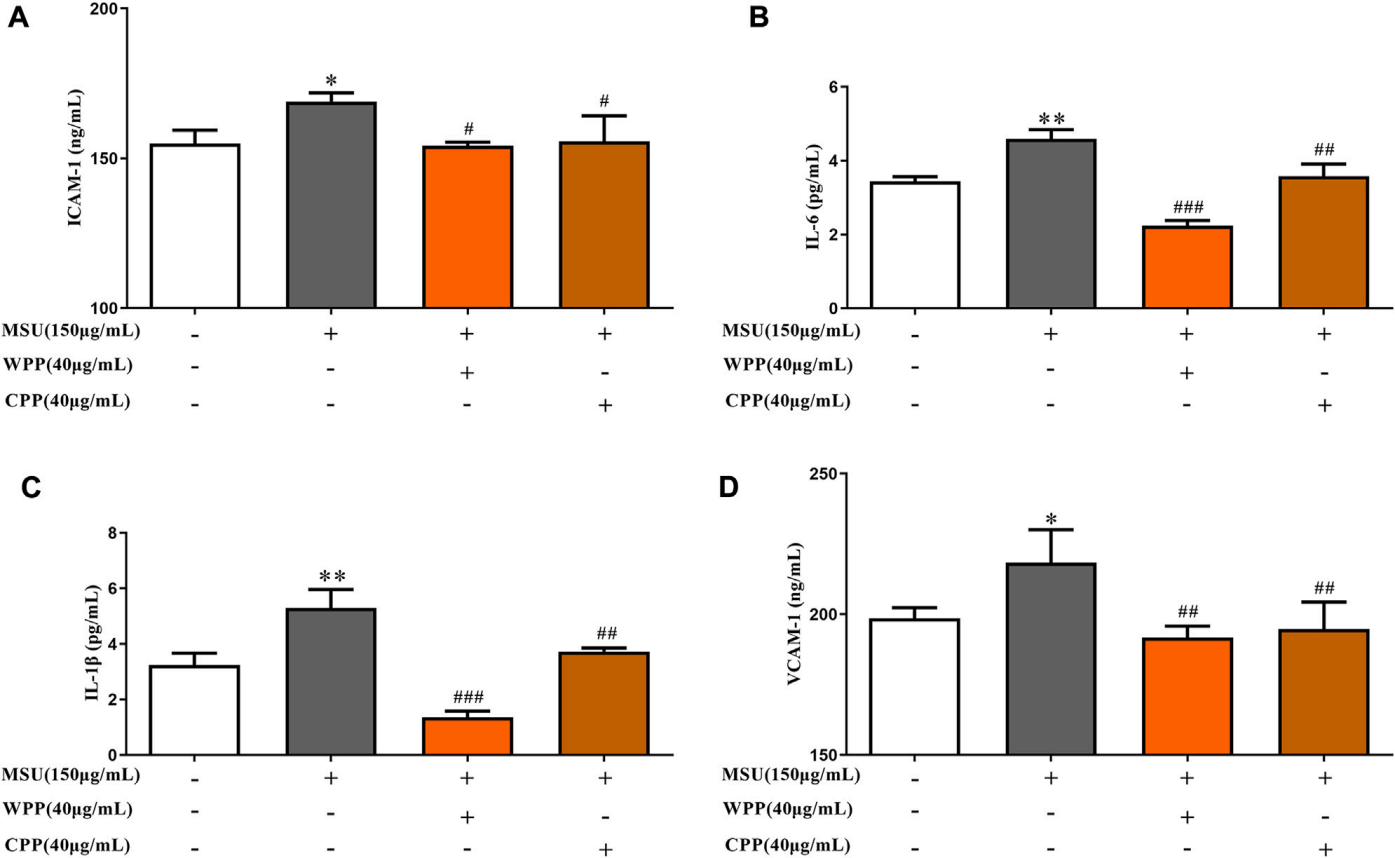
Effects of WPP and CPP on ICAM-1 **(A)**, IL-6 **(B)**, IL-1β **(C)** and VCAM-1 **(D)** in the *in vitro* gout model, *p < 0.05, **p < 0.01 vs. control group; ^#^ *p* < 0.05, ^##^ *p* < 0.01, ^###^ *p* < 0.001 vs. model group; *n* = 3.

As shown in Figure 3, compared with the control group, the ICAM-1 (*p* < 0.05), VCAM-1 (*p* < 0.05), IL-1β (*p* < 0.01) and IL-6 (*p* < 0.01) contents in the model group were significantly increased, suggesting that MSU induces inflammatory injury in HUVECs. Compared with the model group, the ICAM-1 (*p* < 0.05), VCAM-1 (*p* < 0.01), IL-1β (*p* < 0.001) and IL-6 (*p* < 0.001) contents were significantly decreased after treatment with 40 μg/ml WPP and MSU. The ICAM-1 (*p* < 0.05), VCAM-1 (*p* < 0.01), IL-1β (*p* < 0.01) and IL-6 (*p* < 0.01) contents were also significantly decreased after treatment with 40 μg/ml CPP and MSU. The results indicated that both WPP and CPP could alleviate MSU-induced inflammatory injury by downregulating the expression of ICAM-1, VCAM-1, IL-1β and IL-6.

### WPP and CPP can reduce the expression of TLR4 and NLRP3

**FIGURE 4:**
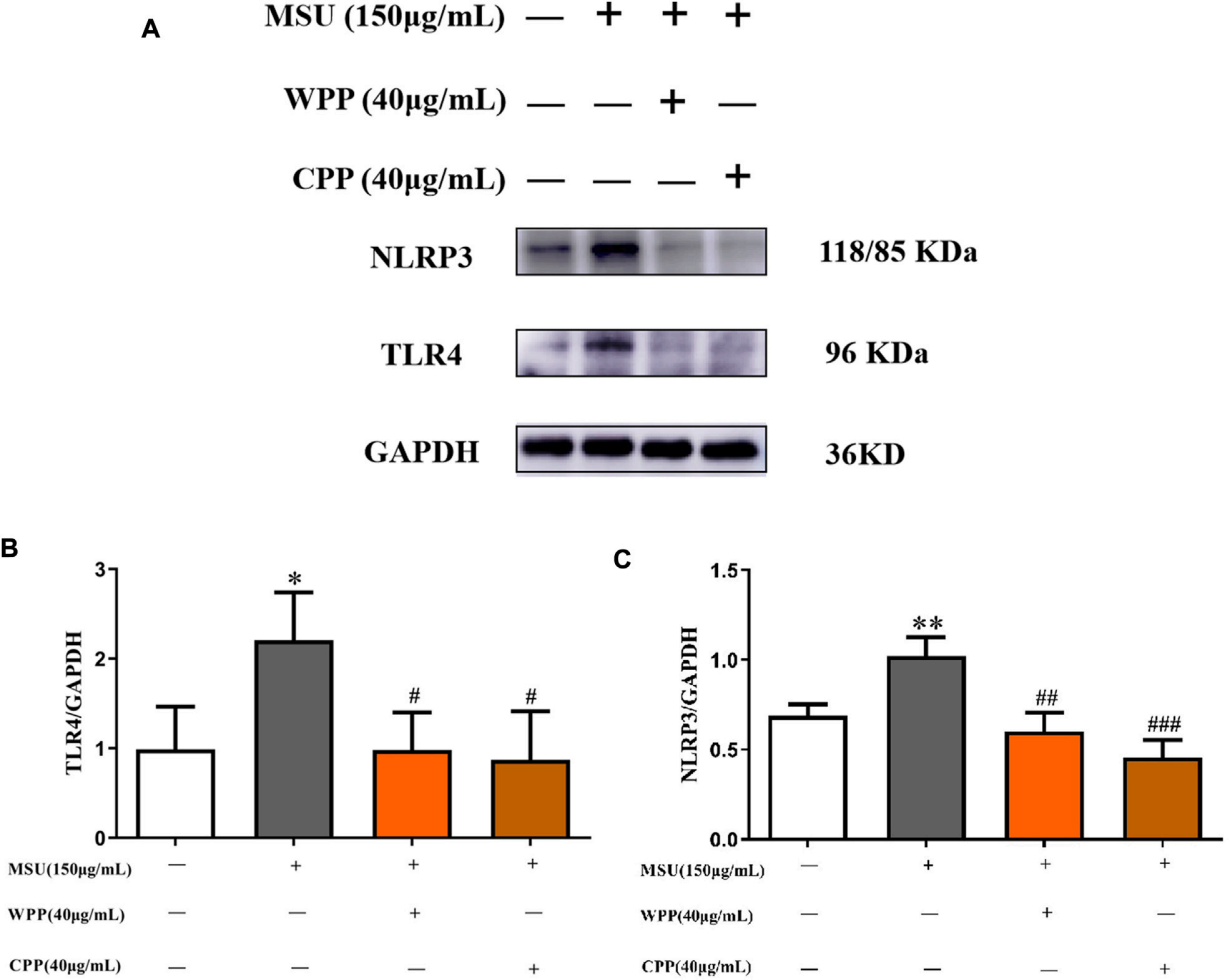
Western blotting was used to detect the effect of WPP and CPP on the protein expression of TLR4 and NLRP3 **(A)**, and ImageJ was used to measure the gray value of the protein bands **(B,C)**, *p < 0.05, **p < 0.01 vs. the control group; ^#^ *p* < 0.05, ^##^ *p* < 0.01, ^###^ *p* < 0.001 vs. the model group; *n* = 3.

As shown in Figure 4, compared with the control group, the protein expressions of TLR4 (*p* < 0.05) and NLRP3 (*p* < 0.01) in the model group were significantly increased, indicating that the NLRP3 inflammatory corpuscle pathway recognized and mediated by TLR4 was activated. After the 40 μg ml WPP treatment, the protein expressions of TLR4 (*p* < 0.05) and NLRP3 (*p* < 0.01) were significantly decreased. At 40 μg/ml, CPP also significantly downregulated the protein expression of TLR4 (*p* < 0.05) and NLRP3 (*p* < 0.001).

### WPP and CPP can inhibit ROS

**FIGURE 5:**
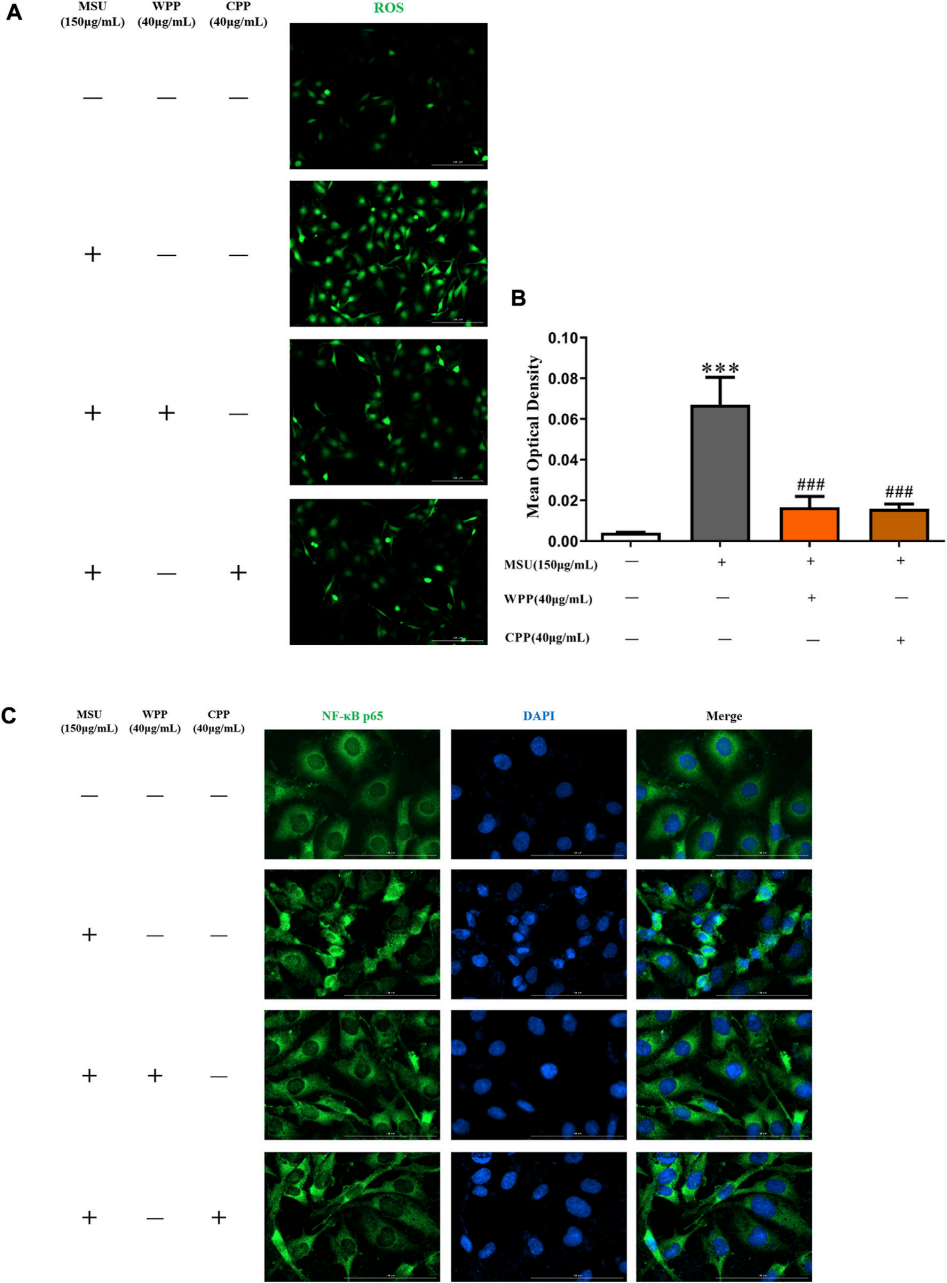
The generation of ROS was compared by IF **(A)**, scale bar = 200 μm, and the fluorescence intensity was quantitatively analyzed **(B)**, ***p < 0.001 vs. the control group; ^###^ *p* < 0.001 vs. the model group; *n* = 3. Effect of WPP and CPP on NF-κB p65 nuclear translocation by IF **(C)**.

As shown in Figures 5A,B, compared with the control group, the ROS content in the model group was significantly increased after MSU stimulation (*p* < 0.001), indicating that the intracellular oxidative stress response was activated. Compared with the model group, 40 μg/ml WPP or CPP cotreated with MSU for 24 h significantly inhibited ROS production (*p* < 0.001), indicating that oxidative stress injury was alleviated and inhibited. Therefore, WPP and CPP can alleviate MSU-induced oxidative stress injury by inhibiting ROS generation.

### WPP and CPP inhibited NF-κB activation

In the control group, NF-κB p65 was mainly expressed in the cytoplasm, and there was no obvious nuclear entry, as fluorescent nuclear division was clearly observed. After MSU stimulation, the nuclear translocation of NF-κB p65 in the model group was significantly increased, there was no obvious nuclear-cytoplasmic division indicated by fluorescence, and the nuclear green fluorescence intensity was significantly enhanced, indicating that the NF-κB pathway was activated and had triggered downstream inflammatory reactions. Compared with the model group, the nuclear translocation of NF-κB p65 was significantly improved after treatment with 40 μg/ml WPP or CPP and MSU for 24 h, and fluorescent nuclear division was enhanced. The above results showed (Figure 5C) that WPP and CPP could inhibit activation of the NF-κB pathway by inhibiting NF-κB p65 translocation into the nucleus, thereby alleviating the inflammatory response induced by MSU.

### UHPLC-QE-MS for cell metabolomics analysis

**FIGURE 6:**
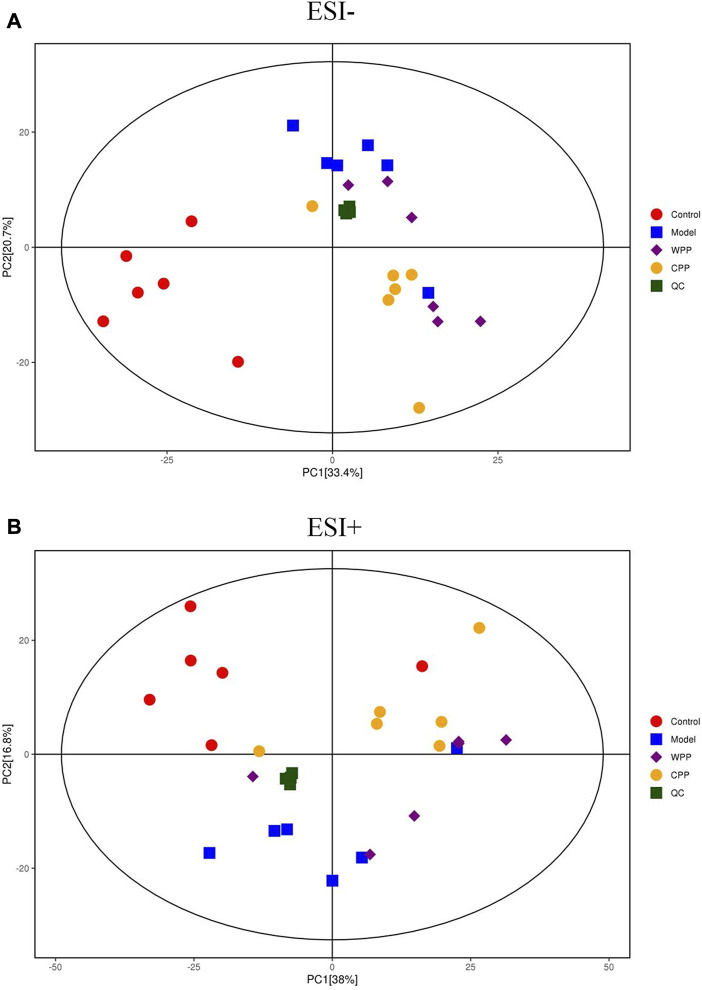
Principal component analysis (PCA) score plot of the control group, model group, WPP-treated group (40 μg/ml) and CPP-treated group (40 μg/ml) in the negative ion mode (NEG) **(A)** and positive ion mode (POS) **(B)** (*n* = 6).

**FIGURE 7:**
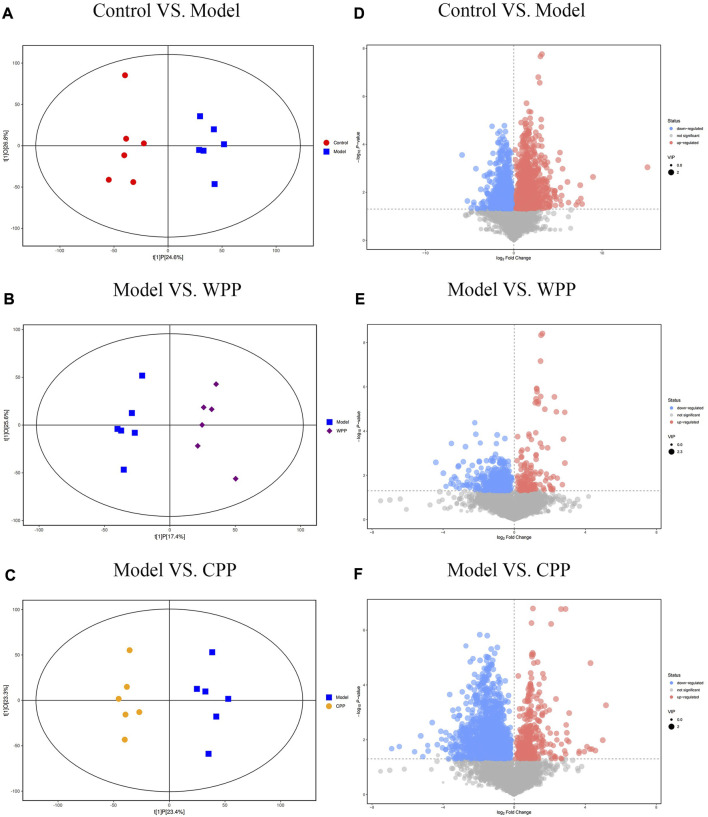
Orthogonal projections for the latent structures-discriminate analysis (OPLS-DA) score plot and volcano plot between the control group, model group, WPP-treated group (40 μg/ml), and CPP-treated group (40 μg/ml) in the positive ion mode (POS) (n = 6). OPLS-DA score plots between the control group and model group **(A)**, model group and WPP-treated group (40 μg/ml) **(B)**, and model group and CPP-treated group (40 μg/ml) **(C)**. The volcano plots between the control group and model group **(D)**, model group and WPP-treated group (40 μg/ml) **(E)**, and model group and CPP-treated group (40 μg/ml) **(F)** are also shown above. The *X*-axis and *Y*-axis represent the log2-fold change and −log_10_ *p* value, respectively.

PCA was used to evaluate the treatment of gout with WPP (40 g/ml) and CPP (40 g/ml) to the control group and the model group to determine changes in the metabolic spectrum. According to the PCA score (Figure 6), there were some differences between the metabolite spectra of the four groups in ESI+ and ESI. Additionally, the OPLS-DA diagram (Figures 7A–C) demonstrated that there may be a substantial difference between the control group, model group, WPP treatment group, and CPP treatment group. Since the database contains more data about chemicals in ESI + than in ESI-, differential metabolites between the groups were screened using a *p* < 0.05 (Student’s t test) and a VIP >1 in ESI+. Specifically, there were 90 upregulated metabolites and 16 downregulated metabolites between the control group and the model group (Figure 7D), 25 downregulated metabolites between the model group and the WPP treatment group (Figure 7E), and upregulated metabolites between the model group and the CPP treatment group (Figure 7F).

**FIGURE 8:**
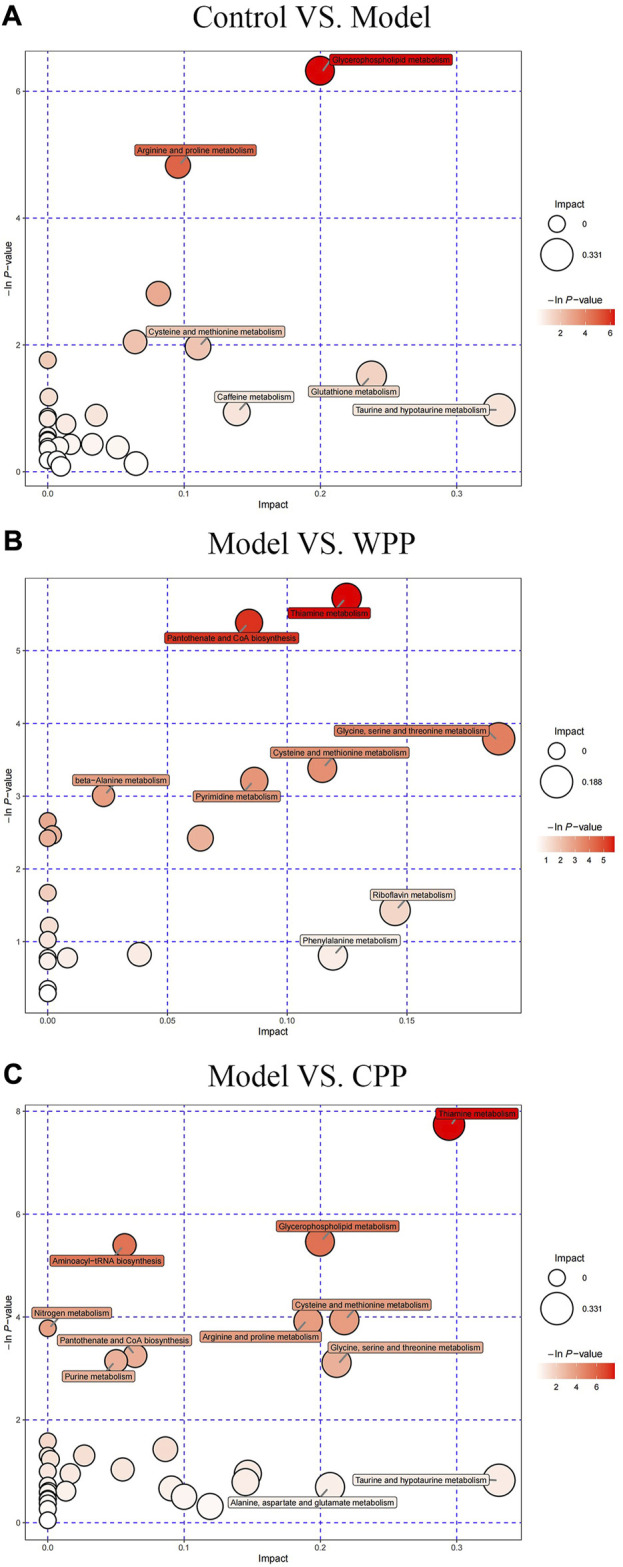
Metabolic pathway bubble plots of the control group and model group **(A)**, the model group and WPP-treated group (40 μg/ml) **(B)**, and the model group and CPP-treated group (40 μg/ml) **(C)**. The *X*-axis and *Y*-axis represent the impact and −ln *p* values, respectively.

Finally, according to the topological analysis, the method of determining the path affects the value (ln *p*) generated in the flow analysis. Therefore, the topological analysis method determines a higher level of metabolic path and is displayed in the bubble diagram. The results (Figure 8) showed that the biosynthesis of glycerophosphate, thiamine, pantothenic acid and coenzyme a may lead to the production and development of uric acid. These altered metabolites were discovered to play a major role in the effects of the treatment on gout, specifically in the phenylalanine metabolism of WPP and the lysophosphatidylcholine metabolism of CPP.

## Discussion

*P. igniarius* is a traditional Chinese herbal medicine that has a long history of application in China. In modern times, it has gradually become the focus of attention in China and abroad because of its outstanding regulation of immunity and its antitumor and antioxidant activities. A series of studies have shown that *P. igniarius* has antiviral (H Dogan et al., 2018), anti-inflammatory (Nakayama et al., 2014), antioxidant (Kottgen et al., 2013), antineoplastic (Li et al., 2015), and immunoregulatory (Li et al., 2015) pharmacological effects, whereas documentation of the anti-uric acid activity of *P. igniarius* is still rare. This study focused on oxidative stress, NF-κB signal transduction and NLRP3 inflammation to study the molecular mechanism of *P. igniarius* on gout-related inflammation caused by MSU crystals in HUVECs. We have proven that *P. igniarius* is an effective oxidative scavenger that plays a key role in regulating the NF-κB pathway and NLRP3 inflammatory bodies through ROS generation during MSU crystal-induced inflammation.

However, the shortage of wild *P. igniarius* resources and the high market prices have indirectly limited the in-depth study of the medicinal value of *P. igniarius* and restricted the development and application of *P. igniarius*. Therefore, artificial cultivation is an important way to efficiently obtain fruiting bodies of *P. igniarius*, and research on the cultivation of *P. igniarius* will effectively alleviate this situation. Our research team has analyzed and studied the main active components of wild and cultivated *P. igniarius*, and the results showed that wild and cultivated samples were similar in composition (Li et al., 2021). Therefore, we used wild and cultivated *P. igniarius* to prepare WPP and CPP, respectively, to study their efficacy and mechanism *in vitro.*

ROS are crucial bioactive mediators that act as second messengers in signal transduction during the pathogenesis of MSU crystal-induced inflammation (Cheng et al., 2015; Choe et al., 2015). Inhibition of ROS production is an important indicator that can be used to evaluate the anti-inflammatory effect of drugs and can demonstrate the antioxidant capacity of drugs (Ren et al., 2020). The antioxidant capacity of known drugs can be determined by the DPPH, FRAP and ABTS methods. In 293 cells derived from the human embryonic kidney, ascorbic acid (Vc), which has antioxidant characteristics and functions as a powerful ROS scavenger, inhibits the production of ROS (Ozgen et al., 2006); Vc was used as a positive control. This study showed that the total polyphenol extracts (WPP and CPP) from *P. igniarius* had good antioxidant capacity, which could theoretically effectively inhibit the production of ROS and have certain anti-inflammatory effects. This study also demonstrated that the total polyphenol content in the prepared WPP and CPP was high.

XO is an enzyme that produces uric acid in the body. Evolutionarily, primates lack uric acid oxidase, and thus they are prone to hyperuricemia and gout (Zulueta et al., 2009; Neogi, 2010). Therefore, XO is the target of therapeutic drugs for hyperuricemia and gout (Nishino, 1994; Hille, 1996). Allopurinol is a commonly used drug in clinical practice for the treatment of gout (Elion, 1966). In this study, WPP and CPP showed similar XO inhibitory effects in a concentration-dependent manner. This suggests that *P. igniarius* may be a potential XO inhibitor.

The main pathological trait of gout is endothelial activation brought on by IL-1 and IL-6 while inducing ICAM-1 expression, which increases the influx of neutrophils into the joint fluid and the subsequent influx of monocytes. Following activation, monocytes and neutrophils actively phagocytose MSU crystals, which sets off inflammatory reactions (Cronstein and Terkeltaub, 2006; Shi et al., 2013; Han et al., 2021). In this study, the ELISA experiment showed that WPP and CPP can inhibit the expression of IL-1β, IL-6, ICAM-1 and VCAM-1 to inhibit the phagocytosis of MSUs. The expression of the inflammasome (TLR4 and NLRP3) is associated with acute gout inflammation (Kim et al., 2016; Zhang et al., 2022), which was determined by western blot, and WPP and CPP inhibited the expression of TLT4 and NLRP3. Thus, *P. igniarius* further inhibited the occurrence of acute gout inflammation. This study proves that *P. igniarius* is an effective drug for gout. Moreover, WPP and CPP have similar effects at the same concentration. This suggests that high-priced WPP can be replaced with low-priced CPP.

The NF-κB signaling pathway is an important signaling pathway regulating inflammation *in vivo*. Its activation is determined by the nuclear entry of NF-κB p65. Immunofluorescence results showed that WPP and CPP could inhibit NF-κB p65 entry into the nucleus and alleviate the inflammatory response induced by MSU, which is consistent with our previous conclusion.

**FIGURE 9:**
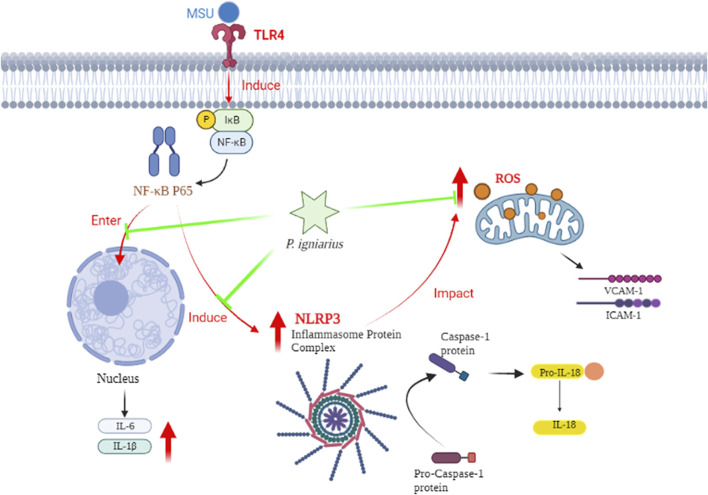
The hypothetical mechanism of *P. igniarius* in HUVECs.

In conclusion, we can speculate on the mechanism of *P. igniarius*. MSU crystals induce inflammasome maturation and inflammatory factor secretion (IL-1, IL-6, ICAM-1, and VCAM-1) in HUVECs through activating the NF-κB pathway and producing ROS. *P. igniarius* reduced the production of ROS and NF-κB activation, which in turn prevented the activation of inflammasomes (Figure 9).

Metabolomics has made significant advances in helping to understand the pathogenesis of many metabolic diseases, which has proven the validity of metabolite profiling (Shan et al., 2021). Various methods have been conducted to explore effective treatments for hyperuricemia such as magnetic resonance spectroscopy and liquid and gas chromatography-tandem mass spectrometry (Wang et al., 2020). Therefore, metabolomics based on UHPLC-QE-MS, which is suitable for the simultaneous and systemic analysis of multiple metabolite fingerprinting, was employed to generate metabolite profiles of cells to characterize the metabolic changes related to hyperuricemia and uncover the underlying metabolic mechanism of the therapeutic effects of WPP and CPP on gout. In our study, it was found that the *in vitro* model of MSU-induced gouty arthritis in HUVECs was mainly related to lysophosphatidylcholine metabolism and phenylalanine metabolism. Studies have shown that lysophosphatidylcholine (lysoPC) is the end product of glycerophospholipid metabolism. Abnormal levels of lysoPC can lead to lipid peroxidation damage and inflammation (van der Veen et al., 2017). From the biomarkers found in metabolomics, the expression level of lysoPC in the model group was upregulated, whereas lysoPC in the WPP and CPP groups was downregulated. Therefore, WPP and CPP may treat gouty arthritis by affecting the metabolism of lysoPC. However, phenylpyruvate, a dimethyl compound, is a product of phenylalanine metabolism, and several studies have reported that hyperuricemia and gout are related to phenylalanine metabolism (Liu et al., 2012; Jiang et al., 2017). Phenylpyruvate can further generate phenylacetaldehyde, which generates phenylacetaldehyde-CoA, which is related to pantothenate and CoA biosynthesis through acetyl-CoA (Wu et al., 2018). According to our research results, the expression of l-phenylalanine in the model group was upregulated, whereas those in the WPP and CPP groups were downregulated.

## Conclusion

In conclusion, we showed that both wild and cultivated *P. igniarius* have anti-gout arthritis properties; their similar active ingredients and pharmacological properties are anticipated to encourage the development and use of cultivated *P. igniarius* to fill the void left by wild *P. igniarius*. In the future, we’ll concentrate on the targets and pathways involved in *P. igniarius*’s treatment of gout (Dalbeth et al., 2016).

## Data Availability

The raw data supporting the conclusions of this article will be made available by the authors, without undue reservation.

## Glossary

DPPH: 2,2-diphenyl-1-(2,4,6-trinitrophenyl) hydrazyl stable free radical assay
ABTS: 2,2-azino-bis-3-ethylbenzothiazoline-6-sulfonic acid assay
FRAP: Ferric Reducing/Antioxidant Power assay
IR: Inhibition rate
WPP: Wild P. igniarius total polyphenols
CPP: Cultivated P. igniarius total polyphenols
XO: Xanthine Oxidase
MSU: Monosodium urate
ICAM-1: Intercellular adhesion molecule-1
IL-1β: Interleukin-1β
IL-6: Interleukin-6
TNF-α: Tumour necrosis factor
VCAM-1: Vascular cell adhesion molecular-1
TLR4: Toll like receptor 4
NLRP3: NOD-like receptor thermal protein domain associated protein 3
ROS: Reaction oxygen species
NF-κB p65: Nuclear factor-κB p65
NF-kB: Nuclear factor-κB
IκBα: Nuclear factor-κB inhibitor protein
NSAIDs: Nonsteroidal anti-inflammatory drugs
Vc: Ascorbic acid
lysoPC: Lysophosphatidylcholine
IC_50_: Half-maximum inhibitory concentration
DMSO: Dimethyl sulfoxide
OD: Optical density
DCFH-DA: dichloro-dihydro-fluorescein diacetate
LC-MS/MS: Liquid chromatography-tandem mass spectrometric
IDA: Information-dependent acquisition
PCA: Principal component analysis
OPLS-DA: Orthogonal projections to latent structures-discriminate analysis
SD: Standard deviation
Vc: Vitamin C
XO: Xanthaxanthin oxidase
PVDF: Polyvinylidene fluoride
ECL: Enhanced chemiluminescence
QC: Quality control
UHPLC-QE-MS: Ultra-high-performance liquid chromatography/Q Exactive HF-X Hybrid Quadrupole-Orbitrap Mass
IDA: Information-dependent acquisition
PCA: Principal component analysis
OPLS-DA: Orthogonal projections to latent structures-discriminate analysis
VIP: Variable importance in the projection
ANOVA: Analysis of variance
MTT: Methyl thiazole tetrazolium
PCA: Principal component analysis
ELISA: Enzyme-linked immunosorbent assay
POS: Positive ion mode
NEG: Negative ion mode
